# A Chemometric Approach to Oxidative Stability and Physicochemical Quality of Raw Ground Chicken Meat Affected by Black Seed and Other Spice Extracts

**DOI:** 10.3390/antiox9090903

**Published:** 2020-09-22

**Authors:** Małgorzata Muzolf-Panek, Anna Kaczmarek, Jolanta Tomaszewska-Gras, Renata Cegielska-Radziejewska, Tomasz Szablewski, Małgorzata Majcher, Kinga Stuper-Szablewska

**Affiliations:** 1 Department of Food Quality and Safety Management, Faculty of Food Science and Nutrition, Poznań University of Life Sciences, Wojska Polskiego 31, 60-637 Poznań, Poland; anna.kaczmarek@up.poznan.pl (A.K.); jolanta.tomaszewska-gras@up.poznan.pl (J.T.-G.); renata.cegielska-radziejewska@up.poznan.pl (R.C.-R.); tomasz.szablewski@up.poznan.pl (T.S.); 2 Department of Food Chemistry and Instrumental Analysis, Faculty of Food Science and Nutrition, Poznań University of Life Sciences, Wojska Polskiego 31, 60-637 Poznań, Poland; malgorzata.majcher@up.poznan.pl; 3 Department of Chemistry, Faculty of Wood Technology, Poznań University of Life Sciences, Wojska Polskiego 75, 60-625 Poznań, Poland; kinga.stuper@up.poznan.pl

**Keywords:** black seed, cloves, allspice, spice extracts, lipid oxidation, protein oxidation, raw chicken meat, microbiological analysis, chemometric analysis

## Abstract

The effects of black seed (*Nigella sativa*), allspice, bay leaf, caraway, cardamom, clove, and nutmeg extracts on the quality of raw ground chicken legs stored at 4 °C were investigated. During 12 days of storage, conjugated diene (CD) content, thiobarbituric acid reactive substances (TBARS), oxidation induction time (IP) by DSC (differential scanning calorimetry), hexanal content by GC-SPME-MS, thiol group (SH) content were determined. Moreover, microbial growth, pH and color of the samples were investigated. Sensory analysis was also realized. All extracts increased oxidative stability and safety of meat, significantly changed the color of the samples, stabilized the pH and increased their sensory scores (except color of samples with bay leaf and black seed) when comparing to control. Black seed, allspice and clove extracts showed high antioxidant capacity in lipid (CD = 0.23%, 0.28%, and 0.37%, respectively; TBARS = 0.55, 0.50, and 0.48 mg/kg, respectively) and protein fraction (SH content = 47.9, 52.1 and 52.7 nmol/g, respectively), although the ABTS^•+^ radical scavenging activity of black seed (33.1 µM/g) was significantly lower than the cloves (2496 µM/g) and allspice (815 µM/g). In the sensory analysis the highest scores were ascribed to the sample with cardamom followed by cloves. Principal component analysis (PCA) indicated complex and inseparable interrelationship among lipid and protein oxidation processes and the relationship of the protein oxidation on the lightness of meat. The results enabled to discriminate the meat samples, showing a great impact of the extracts on the final quality of raw chicken meat with black seed being potent antioxidant active additive.

## 1. Introduction

Poultry meat is the most consumed meat worldwide, and the second, after pork, in countries of the EU [1]. It gained a great popularity among consumers because of its low preparation time necessary, low cost, and a wide offer of easy-to-prepare poultry-based products [2]. Moreover, qualitative and quantitative nutritional characteristics of poultry meat have still increased its consumption on the global market. Chicken meat is an excellent source of highly-digestible proteins with essential amino acid content, lipids with high polyunsaturated fatty acid content, as well as B group vitamins and some minerals [3,4]. Nevertheless, the much higher degree of unsaturation of fatty acids of chicken meat in comparison to other types of meat accelerates the oxidative processes, leading to the fast decrease of meat quality [4,5]. Lipid oxidation causes deteriorations in meat flavor, color, physicochemical properties, nutritional value, and finally food safety [4,6]. Other factors affecting oxidative stability of meat are storage conditions and technological processes the meat is subjected to, like grinding which increases meat surface exposed to reactive oxygen species (ROS) [7]. Additionally, chicken meat is prone to the contamination of undesired microorganisms originating from animal, abattoir facilities, manufacturing and/or storage. Gram positive: Lactic acid bacteria (LAB) and Gram negative: *Pseudomonas* and *Enterobacteriaceae* are the most common bacteria in raw meat related with its spoilage [8].

Herbs and spices have been used since ages not only for meat seasoning to give a specific flavor to the final products but also to delay the spoilage of meat during storage. At present, the positive effect of plants and plant extracts on meat quality has been ascribed to their high antioxidant and antimicrobial activities [5,9,10,11,12,13]. The trend of using natural agents as food preservatives has been still of interest since the consumers, especially in EU and US countries, demand for “clean label” in meat product.

Hardly any study has been conducted to investigate the efficiency of black seed extract in muscle food system [11,13]. Black seed and spices like allspice, bay leaf, cardamom, caraway, cloves, and nutmeg are commonly used in Asian and European kitchen. Their antioxidant properties have been applied to protect meat products from oxidation [9,11,13]. Nevertheless, a direct comparison of their effectiveness in meat is difficult due to the large variety of methods employed to prepare the extracts and to measure their antioxidant activity in the meat matrix [14,15].

Thus, the aim of this study was to investigate the effect of black seed and allspice, bay leaf, cardamom, caraway, clove, and nutmeg extracts on the oxidative quality of raw ground chicken legs stored at 4 °C. According to the best knowledge of authors the effect of black seed (*Nigella sativa*) addition to chicken meat and the comparison of its properties to other spices had been investigated for the first time.

## 2. Materials and Methods

### 2.1. Chemicals

2,2′-azinobis-(3-ethylbenzothiazoline-6-sulfonic acid) (ABTS), Ellman’s reagent—5,5′-dithiobis(2-nitrobenzoic acid) (DTNB), sodium dodecyl sulfate (SDS), 6-hydroxy-2,5,7,8-tetramethylchroman-2-carboxylic acid (Trolox), 2-thiobarbituric acid (TBA), 1,1,3,3-tetramethoxypropane, phenolic compounds and organic solvents of HPLC grade were purchased from Sigma-Aldrich (Steinheim, Germany). All other chemical reagents and solvents used in this study were analytical grade and obtained from POCh (Gliwice, Poland). All agars for microbiological analyses were purchased from Yongxin Biological Technology Co., Ltd. (Yixing, Jiangsu, China).

### 2.2. Materials

Dried spices (allspice, bay leaf, black seed, cardamom, caraway, cloves, and nutmeg) were purchased from local distributor (Ciecierzyn, Polska). Chicken legs, provided by a local producer of meat (Swarzędz, Poland), were deboned and the meat was cut and minced through a 5 mm plate on the place. Then, the meat was transported to the laboratory in the insulated iceboxes (to avoid microbial contamination), in chilled condition (4 °C) within half an hour.

### 2.3. Preparation of Spice Extracts

The spices were grounded and 15 g of each one was extracted with 225 mL of 50% aqueous ethanol in a closed container for 24 h on the magnetic stirrer in the dark. The extraction conditions were chosen based on the preliminary study with various concentrations of ethanol in water (0%, 50% and 100%) and time of the process (from half an hour to 24 h) [16]. After filtration through 3HW Filtrak filter paper (Filtrak, Niederschlag Bärenstein, Germany) the antioxidant activity and phenolic content of the extract were analyzed. The results on the DPPH^•^ (diphenyl picrylhydrazyl) radical scavenging activity, ferric reducing antioxidant power (FRAP) and total phenolics were presented and discussed in our previous paper [13]. To prepare meat sample the spice extracts were freeze dried.

### 2.4. ABTS^•+^ Radical Scavenging Capacity of Spice Extracts

The antioxidant activity of spice extracts could be determined by methods, which reflect the various mechanisms of antioxidant action including the scavenging of free radicals (DPPH^•^ and ABTS^•+^) or chelation of transition metal ions (FRAP method). The results on the DPPH^•^ radical scavenging activity and FRAP were presented previously, thus this paper includes the results of TEAC assay.

The radical scavenging activity of extracts were assessed by the TEAC (Trolox Equivalent Antioxidant Capacity) assay [17]. The ABTS^•+^ radical cation was generated with potassium disulphate according to the modifications of [18]. Briefly, 7 mM ABTS in water was mixed with 2.45 mM potassium disulphate at a ratio of 2:1. To produce radicals the mixture was incubated in dark at room temperature for 12–16 h. Then, the ABTS^•+^ solution was diluted with PBS (phosphate buffer saline), pH 7.4 to an absorbance of 0.7 (±0.01). 990 µL of ABTS^•+^ solution was mixed with 10 µL of spice extract and, after 6 min of incubation in dark, the decrease in the absorbance of the mixture was read at 734 nm against blank sample (ABTS^•+^ solution with ethanol). The results were expressed as TEAC values in µM Trolox/g dry weight.

### 2.5. UPLC Analysis of Phenolic Compounds in Spice Extracts

Extract samples (0.20 g) were placed in sealed 17 mL culture test tubes, where first alkaline and then acid hydrolysis was run. In order to run alkaline hydrolysis 1 mL of distilled water and 4 mL of 2 M aqueous sodium hydroxide were added to test tubes. Tightly sealed test tubes were heated in a water bath at 95 °C for 30 min. After cooling (during 20 min) test tubes were neutralized with 2 mL of 6 M aqueous hydrochloric acid solution (pH = 2). Next, samples were cooled in water with ice. Flavonoids were extracted from the inorganic phase using diethyl ether (2 × 2 mL). Ether extracts were transferred to 8 mL vials. Then, acid hydrolysis was run. For this purpose, the aqueous phase was supplemented with 3 mL of 6 M aqueous hydrochloric acid solution. Tightly sealed test tubes were heated in a water bath at 95 °C for 30 min. After being cooled in water with ice the samples were extracted with diethyl ether (2 × 2 mL). Produced ether extracts were continuously transferred to 8 mL vials. Then, these extracts were evaporated to dryness under nitrogen stream. Prior to analysis all samples were dissolved in 1 mL of methanol. Analysis was performed using an Aquity H class UPLC system equipped with a Waters Acquity PDA detector (Waters, USA). Chromatographic separation was performed on an Acquity UPLC^®^ BEH C18 column (100 mm × 2.1 mm, particle size 1.7 μm) (Waters, Ireland). The elution was carried out gradient using following mobile phase composition: A: Acetonitryl with 0.1% formic acid, B: 1% aqueous formic acid mixture (pH = 2).

Concentrations of flavonoids were determined using an internal standard at wavelength of 320 nm. Concentrations of phenolic acids were determined using an internal standard at wavelength of 280 nm. Compounds were identified based on a comparison of retention times of the analyzed peaks with the retention times of the standards and by adding a specific amount of the standards to the analyzed samples and a repeated analysis. Detection level was 1 μg/g.

### 2.6. Preparation of Meat Samples

Freeze dried extract (prepared as above) was dissolved in 60 mL of water (to better spread out the extract in the matrix) and mixed with meat (3 kg) for 3 min (each extract was mixed separately). The final concentration of the spice in meat was 0.5% (*m/m*). The following samples were prepared from meat: Control (meat mixed with 60 mL of water) and 7 treatments: Allspice (A), bay leaf (BL), black seed (BS), cardamom (CD), caraway (CR), cloves (CL), and nutmeg (N). Then, each sample of 100 g was packed in a low-density polyethylene bag and kept at 4 °C (±1 °C) for further analyses, performed on 0, 3rd, 5th, 7th, 10th, and 12th days of storage. Total number of samples of one batch for physicochemical analyses was 144.

### 2.7. Extraction of Lipid Fraction from Meat Samples

Extraction of fat from meat samples was performed by the method of [19]. The chloroform was evaporated from fat samples under a nitrogen stream and the samples were stored at −20 °C for further analysis of CD concentration and determination of IP values by DSC.

### 2.8. Lipid Oxidation Analysis

#### 2.8.1. Analysis of CDs

CD concentration was measured by the Cary 1E spectrophotometer using the Ti 1a-64 method [20]. Absorbance of the sample was read at the wavelength of 233 nm against blank (isooctane). CD content was calculated according to the formula:

CD = 0.84 × [(a/b × c) − K_0_] (1)

where a is the absorbance, b is the cuvette length (cm), c is the sample concentration in isooctane (g/L), and K_0_ represents the absorptivity by acid or ester groups (0.07 for esters, 0.03 for acids) [13].

#### 2.8.2. Determination of TBARS

TBARS were determined by the method of [21] with some modification [10]. Ten grams of sample was homogenized (15,000 rpm, 30 s) with 30 mL of aqueous trichloroacetic acid (7.5%). After filtration, 5 mL of the sample was mixed with 5 mL of 0.02 M TBA and put in water bath at 100 °C for 35 min. Then, the sample was cooled and the absorbance was read at 532 nm by the Cary 1E spectrophotometer against the blank sample (5 mL of distilled water with 5 mL of TBA). The TBARS values were calculated from the standard curve of MDA (malonodialdehyde) which was prepared from 1,1,3,3-tetramethoxypropane and expressed in mg of MDA per kg of meat.

#### 2.8.3. Hexanal Content

Hexanal concentration was determined according to previously described method [22] with minor changes [13]. For isolation of volatiles CAR/PDMS/DVB SPME (solid phase microextraction) fiber has been used. Sample preparation required placement of 5 g of chicken meet sample in 20 mL headspace vials with addition of 5 mL of saturated NaCl solution. For quantitation reasons [_2_H^12^]-hexanal (Sigma-Aldrich; Poznań, Poland) as internal standard has been added in the amount to reach 1 mg/kg concentration. Extraction of volatiles was performed automatically using CTC combipal autosampler (Agilent Technologies) at 45 °C during 30 min. Separation and identification of volatiles was performed using gas chromatography and mass spectrometry (GC/MS) by Agilent Technologies 7890A GC coupled to a 5975C MSD with a Supelcowax-10 column (30 m × 0.25 mm × 0.5 µm). Operating conditions for GC/MS were as follows: Helium as a carrier gas with 32.2 cm/s flow; oven temperature program were as follows: 40 °C (1 min), 9 °C/min to 240 °C (3 min). Mass spectra were collected in a scan range of m/z 33–350 with ions sourced at 220 °C and the transfer line heated up to 260 °C. Desorption of volatiles was with splitless injection at 260 °C. The identification of hexanal was performed by a comparison of mass spectra and retention indices (RI) with the 05 NIST library and respective standard (Sigma-Aldrich; Poznań, Poland). The concentration in the sample was calculated from the ratio of the peak area of the hexanal and its corresponding internal labeled standard [_2_H^12^]-hexanal obtained for selected ions: 56 and 64 respectively and corrected with the response factor (Rf = 1.2). The Rf was calculated in the standard mixture of labeled and unlabeled compound in known concentration of 1 mg/kg.

#### 2.8.4. DSC Analysis

The measurements of oxidative stability were carried out with a Perkin-Elmer DSC 7 device (PerkinElmer, Corp., Norwalk, CT, USA) equipped with an Intracooler II and Pyris software 10.0. Nitrogen (99.98% purity) was the purge gas and oxygen (99.999% purity) was the oxidation agent. The DSC calorimeter was calibrated for temperature and enthalpy using indium (m.p. 156.6 °C, ΔHf = 28.45 Jg^−1^) and n-dodecane (m.p. −9.65 °C, ΔHf = 216.73 Jg^−1^). Isothermal determination of oxidation induction period (IP) was performed accordingly to the method described for oxidative stability measurements [23]. The samples of extracted fat of 8–10 mg were weighed into open aluminum pans (Perkin Elmer, No. 02190041, Waltham, MA, USA). At least three samples were analyzed of each fat. The thermal program includes heating from 25 to 140 °C at a scanning rate of 5 °C min^–1^ under nitrogen flow and isothermal heating at 140 °C under oxygen flow of 20 mL/min. The reference was the same open and empty aluminum pan. After normalization of oxidation DSC curve, the IP parameter was determined as the intersection of the extrapolated baseline and the tangent line (leading edge) of the recorded exotherm.

### 2.9. Protein Oxidation

Thiol (sulfhydryl) group (SH) content was determined as a marker of protein oxidation in meat samples. SH content was determined using Ellman’s method [24] and expressed in nmol cysteine equivalent per mg of protein. Two grams of sample was homogenized with 40 mL 0.5% SDS in 100 mM Tris buffer (pH 8.0) for 30 s. Then, the sample was incubated in a water bath (80 °C) for 30 min, cooled and centrifuged during 20 min (3000 rpm). After filtration, 0.5 mL of filtrate was mixed with 2 mL of 100 mM Tris buffer (pH 8.0) and 0.5 mL of 10 mM DTNB in 100 mM Tris buffer and kept for 30 min in dark. The absorbance readings were performed at 412 nm against blank (0.5 mL of 0.5% SDS with 2 mL of 100 mM Tris buffer and 0.5 mL of 10 mM DTNB). Bovine serum albumin was used to prepare the standard curve for protein content determination. To this end the absorbance of the filtrate was read at 280 nm.

### 2.10. Color Measurements

Color was measured on the Konica Minolta CM-5 spectrophotometer in Specular Component Included (SCI) mode, using D65 as light source and 10° standard observer. The automatic calibration (100% calibration) of the instrument was performed at each start using the internal white plate. Zero (0%) calibration was done manually before each analysis. The color was defined in terms of color space values: L* (brightness), a* (redness), and b* (yellowness). Each sample was prepared in triplicates. For one replication, three automatic measurements were performed on a 30 mm diameter surface of the polystyrene plate in which the meat was put.

### 2.11. pH Determination

The pH measurements were carried out by the Elmetron CP- 551 pH meter (Elmetron, Zabrze, Poland) equipped with ERH- 12-6 electrode after calibration. Each sample was prepared in the following procedure: 5 g of meat sample was mixed with 5 mL of distilled water (room temperature) and homogenized during 30 s using Ultra-Turrax T25 homogenizer (16,000 rpm) (IKA, Staufen, Germany).

### 2.12. Microbiological Analysis

The aerobic bacteria, *Enterobacteriaceae* counts, *Pseudomonas* and lactic acid bacteria counts were examined in chicken ground meat. Meat samples (10 g) were homogenized with 90 mL of 0.1% sterile pepton using an Ultra-Turrax T25 homogenizer (IKA, Germany). From an initial suspension of 1:10, serial decimal dilutions were prepared. Bacterial counts were given in CFU per gram and expressed as log10 cfu/g. The microbiological analyses were carried according to the International Organization for Standardization (ISO) reference methods [25,26,27,28].

Standard Plate Count Agar (CM 463, Oxoid, Basingstoke, England) was used to determine total viable counts (TVCs), an indicator of microbial spoilage in the raw ground chicken meat. Incubation was performed at 30 °C for 72 h. *Enterobacteriaceae* counts were identified on a selective VRBG medium (P-0256, BTL, Poland) after incubation at 37 °C for 48 h. Pseudomonas Agar (CM 0559, Oxoid, England) and Pseudomonas CFC Selective Agar Suplement (SR 0103, Oxoid, England) were used to determine the counts of *Pseudomonas*. Incubation was run at 30 °C for 48 h. Lactic acid bacteria counts (LAB) were identified on de Man, Rogosa and Sharpe (MRS) Agar (CM 0361, Oxoid, England). MRS agar was overlaid with a molten medium and incubated at 30 °C for 48–72 h. The confirmation of lactic acid bacteria was done using an oxidase test (MBO 266, Oxoid, England).

### 2.13. Sensory Analysis

Five trained panelists with some experience in sensory evaluation were participated in this study. The evaluation was performed at room temperature in three sessions (replicates) and before each session the samples were stand at room temperature for at least 15 min to equilibrate. Sensory analysis was performed with the quantitative description analysis method (QDA). The panelists assessed three attributes, namely: Odor, color, and texture on the 10 cm unstructured line scale [29,30]. The panel members were asked to put a mark on 10-cm unstructured line scales for each attribute of all the samples. 0 cm is unacceptable characteristic and 10 cm is very acceptable one. The length from the start of the line to marked cross was measured and converted to numerical value for statistical analysis.

### 2.14. Statistical Analysis

The statistical tests were performed using Statistica 13.1 software (StatSoft, Tulsa, OK, USA). The differences were considered significant at *p* ≤ 0.05. Microbiological analysis was performed in eight repetitions. All other tests were run in triplicates. Covariance analysis (ANCOVA) was used to assess the effects of plant additives and storage time on the oxidative and microbiological quality of raw chicken. This analytical procedure is used to consider the effect of the group on the continuous outcome (e.g., TBARS values, SH content), when another continuous explanatory variable (storage time) would also affect the result. Moreover, the one way analysis of variance (ANOVA) was performed (dependent variables: Oxidation markers, physicochemical parameters, sensory analysis scores, microbial count; independent variables: Treatments on each day of storage). The multiple comparison of the meat samples was based on the Duncan’s test to determine the significant differences between samples. Additionally, a Dunnett’s test was performed to compare treatments with a control sample. Also r-Pearson correlation coefficients were calculated (with the *p* values obtained by the Student’s t-test). Finally, PCA and cluster analysis (CA) were used as a first step of data analysis to visualize information and to detect patterns in data, whereas general discrimination analysis (GDA) was used to calculate classification rules for samples discrimination [31]. Selection of variables (importance of variables) for multivariate analysis was performed by the Data Mining tool of Statistic 13.1 based on the Chi-squared values. In all graphs, vertical bars indicate 0.95 confidence interval, and averages with the same superscript on the same day do not differ significantly (*p* > 0.05).

## 3. Results and Discussion

### 3.1. ABTS^•+^ Radical Scavenging Capacity and Phenolic Content of Spice Extracts

The ABTS^•+^ radical scavenging activity of 7 plant extracts is shown in Table 1. Among tested spices, cloves were characterized by the highest antioxidant activity with the TEAC value of 2496 μM/g. The radical scavenging capacity of extracts from allspice and bay leaf was 3-fold and 9-fold lower, respectively, when comparing to cloves. Nutmeg, caraway, black seed and cardamom with the TEAC values ranged from 45 to 13 μM/g formed the group of spices with the lowest antioxidant activity. Our results are in agreement with the study of [32] who showed that the ABTS^•+^ radical scavenging capacity of the extracts from cloves and allspice were very high with the TEAC values of 2071 and 719 μM/g, respectively. In this study, the rank of radical scavenging capacity of extracts provided by the TEAC assay was as followed: Cloves > allspice > bay leaf > nutmeg ≥ caraway ≈ black seed ≥ cardamom and was the same as the rank of DPPH radical scavenging activity and similar to the rank provided by the FRAP assay, both showed in our previous paper [13].

High positive Pearson’s correlation coefficients were observed between the results of TEAC and DPPH methods (r = 0.997 at *p* = 0.000, Student’s t test) and TEAC and FRAP methods (r = 0.998 at *p* = 0.000, Student’s t test). As it was shown previously [13] the antioxidant activity of spice extracts resulted from their phenolic content. For the correlation of ABTS^•+^ radical scavenging activity of spice extracts with their total polyphenol content (from [13]) the r Pearson’s coefficient was 0.988 (at *p* = 0.000, Student’s t test).

Table 2 includes the results of UPLC analysis of phenolic acids and some polyphenols in spice extracts. The highest content of phenolic acids was observed for clove extract. Surprisingly, levels of compounds in allspice extract quantified by UPLC was very low despite showing high total polyphenol content measured by Folin–Ciocalteu’s methods [13]. However, significant correlation was noticed between total phenolic acids by UPLC (Table 2) and the TEAC values (Table 1) of extracts, with r Pearson’s coefficient equaled to r = 0.81 (*p* = 0.027, Student’s t test).

Generally, the direct comparison of phenolic content of extracts with the literature data was difficult, since huge variations were observed in the qualitative and quantitative characteristics of spice extracts reported by others [33,34,35]. These differences could be attributed to the genotypic and environmental differences within species, sample preparation and determination methods.

### 3.2. Lipid Oxidation

#### 3.2.1. Content of CDs

**Figure 1:**
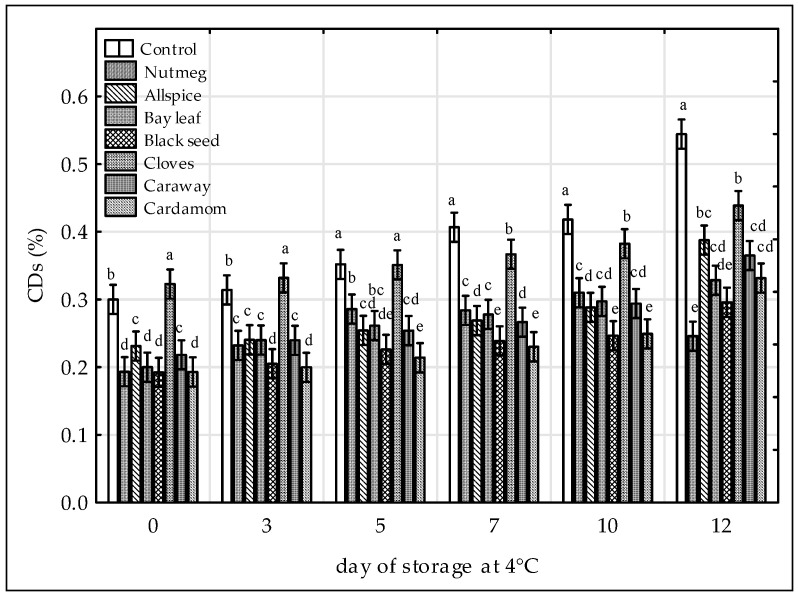
Effect of various spice extract treatments on conjugated dienes (CDs) in raw ground chicken legs during 12 days of storage at 4 °C. Means with the various superscript within the same day are significantly different (*p* ≤ 0.05, Duncan’s test). Vertical bars indicate 0.95 confidence intervals.

Formation of CDs in raw ground chicken legs was highly depended on time and treatments (*p* < 0.05, test F). The CD concentrations of the samples increased with time, reaching the highest values on the 12th day of storage at 4 °C, apart from the sample with nutmeg for which the maximum CD content was observed after 10 days (Figure 1). The results are in agreement with [36], as an increase of CD concentration of the raw chicken patties with mamao luang fruit was observed during 12 days of storage at 4 °C and also [13], reported an increase of CDs during chilled storage of raw ground pork samples. Others indicated that the CD content of the control sample of raw chicken patties was picked on 7th day of storage and dropped thereafter [36] and that the maximum CD concentration in the control sample of cooked chicken thigh was observed on the 2nd day of 5 days of chilled storage [37]. The decrease of CD level at the later stage of storage could be explained by the dissociation of conjugated hydroperoxides to secondary lipid oxidation products of lower molecular weight [36].

In this study, CD concentrations in all treated samples were significantly lower compared to control (Figure 1) with the black seed and cardamom extracts being the most potent antioxidant treatments. For the control sample CD content ranged from 0.30 to 0.54% while for the black seed-treated sample from 0.19% to 0.30% and cardamom-treated sample from 0.19% to 0.33%. The order of samples by increasing content of CD’s was as followed: Black seed ≈ cardamom < nutmeg ≈ bay leaf ≈ caraway ≈ allspice < cloves < control. Others concluded that the inhibition of CD formation by plant extracts was due to their phenolic content [38]. However, in this study the antioxidant effectiveness of spice extracts in meat matrix measured as CD content did not correlate with their antioxidant activity nor with their phenolic contents since black seed and cardamom extracts showed the lowest ABTS^•+^ radical scavenging capacity (Table 1) and phenolic contents among tested samples, as reported previously by [13]. On the other hand, cloves despite the highest ABTS^•+^ radical scavenging activity and phenolic content [13], exhibited the lowest inhibition of CD formation (from 0.32% to 0.44%) in chicken meat among extract tested. For clove treatment, the protective effect on lipid stability was observed from the 7th day of storage (Figure 1). Increased oxidative stability of lipids, expressed as CD marker, was observed in precooked chicken nuggets after addition of grape seed powder [39], cooked chicken thigh containing oregano, sage and honey [37], raw ground pork with persimmon peel extract [38] and raw chicken patties with mamao luang [36]. The data on the beneficial effects of black seed in muscle food system are scarce [11,13]. In our previous study on the oxidative stability of raw ground pork during chilled storage, it was proved that black seed extract reduced CD formation in the higher extent (from 0.07% to 0.16%) than cloves (from 0.11% to 0.17%) and allspice (from 0.10% to 0.20%), when comparing to control (CD values from 0.11% to 0.23%), [13].

The conjugated structures are formed during the first step of lipid oxidation when hydroperoxides are formed from polyunsaturated fatty acids (PUFA) and an electron delocalization on double bond occurs in order to stabilize the radical state [14]. From the comparison of the results of this study to these showed previously [13] it could be observed that the CD levels in chicken meat samples (Figure 1) were higher than that in pork meat samples irrespective to the extract added and time of storage. This is due to the higher content of PUFA in chicken than in pork making chicken meat more prone to the lipid oxidation [4].

#### 3.2.2. TBARS

**Figure 2:**
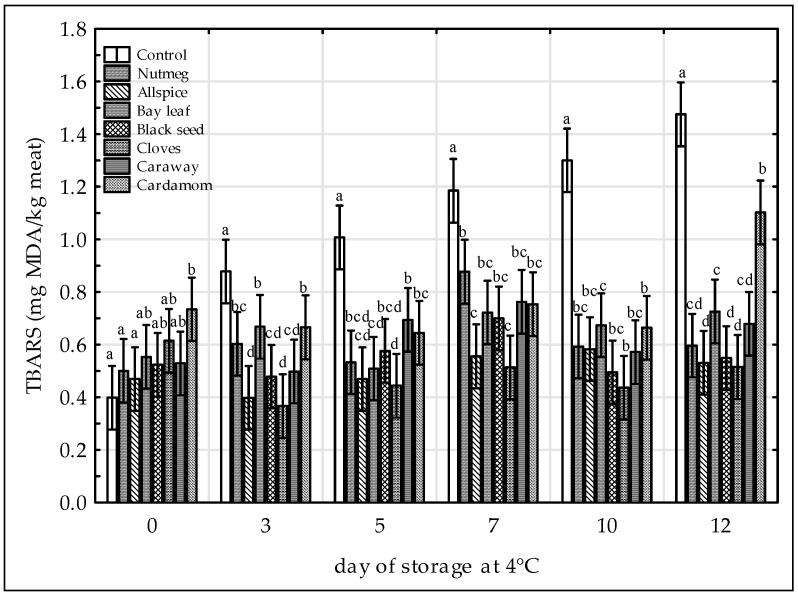
Effect of various spice extract treatments on thiobarbituric acid reactive substances (TBARS) in raw ground chicken legs during 12 days of storage at 4 °C. Means with the various superscript within the same day are significantly different (*p* ≤ 0.05, Duncan’s test). Vertical bars indicate 0.95 confidence intervals.

TBARS values of all samples tested were presented on the Figure 2. Oxidative stability of the samples was significantly affected by the time and treatments (*p* < 0.05, test F). The TBARS values of the control sample stored at 4 °C increased significantly till the end of the period tested, whereas for the samples with the addition of spice extracts TBARS values increased up to 7th day of storage, thereafter decreased slightly and increased again. This could result from the lipid-protein interaction during oxidation processes [40]. The initial TBARS values for the treated samples ranged from 0.47 (allspice) to 0.73 (cardamom) mg MDA/kg and were not significantly different, except cardamom sample, from the control one (0.40 mg MDA/kg) (Figure 2). Except day 0, TBARS values in the control sample were higher than in the samples containing spice extracts on all other analysis days. As shown on the Figure 2 cloves and allspice were the most potent inhibitors of TBARS formation in raw ground chicken meat throughout the storage period which was corroborated by their high ABTS^•+^ radical scavenging activities (Table 1). Black seed was also very effective antioxidant in raw chicken meat, although the extract showed low radical scavenging activity. The inhibitory effects of spice extracts on TBARS formation were as followed: Cloves ≈ allspice ≈ black seed ≥ nutmeg ≈ caraway ≈ bay leaf > cardamom > control. Our findings confirmed the results of other authors who had added various plant extracts and thereby inhibited the development of lipid oxidation in chicken raw meat [9,11,12,13,29,36,41].

Although there was not any legislative limit of TBARS concentration in meat samples, TBARS value over 0.5 mg MDA/kg indicated some oxidation (rancid flavor) and above 1.0 mg MDA/kg as possibly unacceptable level by consumers [42]. Thus, based on the results obtained in this study, clove-treated sample would be perceived as rancid after 12 days, allspice-treated sample after 7 days, and black seed-treated sample after 5 days whereas the control and other samples after 3 days of storage (Figure 2). However, apart from cardamom on 12th day of storage, TBARS values of all treated samples remained under threshold value of 1.0 mg MDA/kg over the storage period. As reported by [9] TBARS values of the raw chicken breast containing aqueous clove extract gained the unacceptable level of 1.0 mg MDA/kg on 6th day of storage. Thus, it could be stated that 50% aqueous ethanol extract of cloves used in this study (Figure 2) was more effective antioxidant agent in raw chicken than the aqueous extract. Moreover, in our previous study [13] almost all treated pork meat samples (except nutmeg-treated sample) were characterized by TBARS values lower than 0.5 mg MDA/kg, irrespective of storage time. This proves that lipid oxidation in chicken meat is more favorable than in pork and that meat matrix plays important role in the extent of the lipid oxidation process. The results of TBARS formation were positively correlated with the results of CD content in chicken meat with the r Pearson’s coefficient of 0.46 (*p* < 0.05, Student’s t test).

#### 3.2.3. Hexanal Content

Among secondary oxidation products, hexanal, a volatile carbonyl compound, has been often used as a marker of 6-fatty acid oxidation [14]. Concentration of hexanal in raw ground chicken legs was shown in Table 3. Both time of storage and extract addition influenced significantly the hexanal content. Except the sample with the addition of black seed, hexanal level increased (up to 3rd day of storage for allspice, cloves, caraway, and cardamom; and up to 7th day for nutmeg and bay leaf) and thereafter a decrease of the values were observed. The effect was probably due to the further oxidation of the compound, as it was previously reported by [21] in cooked turkey meat. The highest hexanal concentrations were observed in the control sample on each storage day with the maximum level of 39.2 mg/100 g after 10 days of storage. The samples treated with black seed and cloves had the lowest hexanal content during the first 3 days. The protective effect of plant extracts on lipid oxidation in meat was also shown by [37] who noticed that the hexanal level increased with time of storage (up to 4th day, at 4 °C) and the addition of oregano and sage extracts to chicken thigh (cooked) inhibited the process. In pork meat, the highest reduction of hexanal formation was observed in the sample containing allspice, then cloves and black seed [13].

#### 3.2.4. DSC Analysis

**Figure 3:**
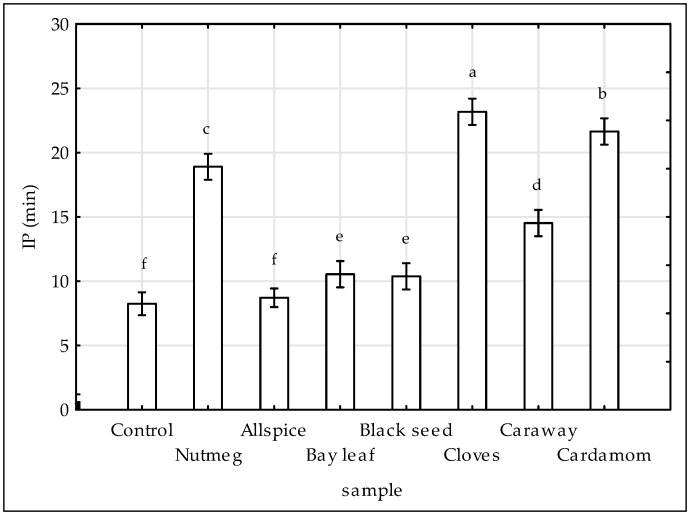
Effect of various spice extract treatments on the oxidation induction period (IP) determined by DSC. Means with the various superscript within the same day are significantly different (*p* ≤ 0.05, Duncan’s test). Vertical bars indicate 0.95 confidence intervals.

The results of DSC analysis were presented on Figure 3. In this study isothermal mode was used to measure IP in minutes. DSC technique makes it possible to study the stability of fats under conditions of elevated temperature, in this case 140 °C, i.e., conditions similar to the thermal treatment of meat in practice. The lower the IP value, the less stable the fat and the lower the antioxidant activity of the extracts. Except allspice treatment, all investigated extracts slowed down lipid oxidation in raw chicken compared to control sample. As shown in Figure 3, the highest efficiency of delaying the oxidation process was demonstrated by extracts of cloves (IP = 23 min), cardamom (IP = 22), and nutmeg (IP = 19 min). It was reported previously by [13] that cloves and nutmeg delayed oxidation of lipid fraction in pork meat. However, in contrast to the results of this study, lipid oxidation in pork meat was accelerated by the extracts of allspice and bay leaf, whereas the addition of black seed, caraway and cardamom to pork meat had no significant effects on the rate of that process [13]. As measured by DSC, lipids from chicken meat with higher PUFA [4] content tend to oxidized faster, than from pork meat (IP = 8 min for chicken control sample, whereas for pork control sample IP = 44 min [13]).

### 3.3. Protein Oxidation

**Figure 4:**
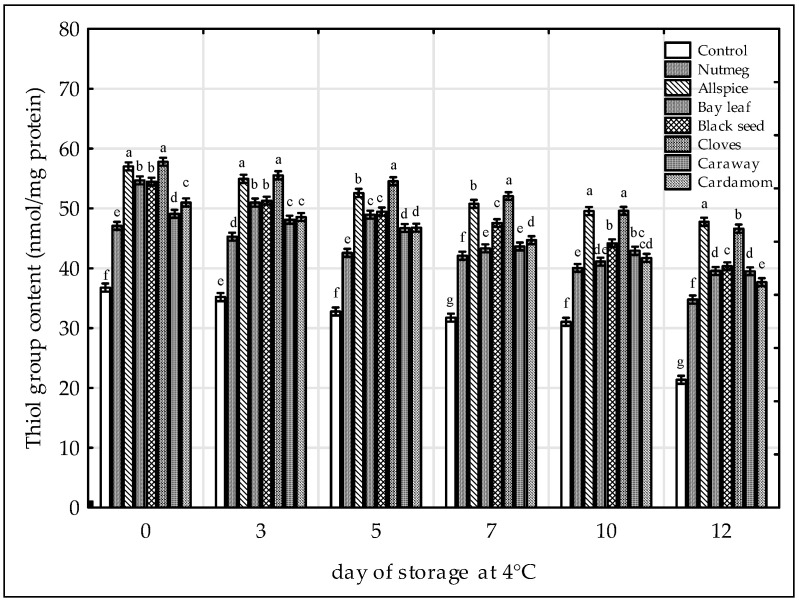
Effect of various spice extract treatments on the thiol group (SH) content in raw ground chicken legs stored at 4 °C. Means with the various superscript within the same day are significantly different (*p* ≤ 0.05, Duncan’s test). Vertical bars indicate 0.95 confidence intervals.

The loss of SH groups, which is a consequence of disulfide formation, has been often used as the marker of protein oxidation [29,38,43]. Based on the results, it could be stated that both, time and the treatments affected significantly the content of SH groups (Figure 4). The loss of SH groups increased in all raw chicken samples when storage proceeded. The lowest content of SH was observed for the control sample (from 36.8 to 21.4 nmol/mg). The addition of extracts increased oxidative stability of proteins in raw chicken legs. The inhibition of disulfide formation was the most pronounced in the samples containing cloves and allspice (mean SH content during storage was 52.2 nmol/mg and 51.6 nmol/mg, respectively). Black seed was also very effective antioxidative treatment (47.4 nmol/mg). The samples could be ranged in the following order of increasing SH group content: Control < nutmeg < cardamom ≈ caraway < bay leaf < black seed < allspice ≤ cloves. Increased oxidative stability of proteins after addition of plant extracts was reported in chicken nuggets stored at −18 °C containing grape seed extract [43], and in raw pork patties with persimmon peel extract [38]. Whereas, in chicken sausages with hydroxytyrosol extract the SH content was lower than in the control sample, which was probably due to protein-phenol interactions [29].

### 3.4. Color and pH

The pH value could be used as the indicator of meat quality which is affected by microbiological changes and various chemical reaction including oxidation. Both factors: Time and treatments had significant effects on the pH values (Table 4). The values of pH of all samples decreased (up to 5th day for control sample and up to 7th day of storage for extract-treated samples), thereafter increased sharply showing the maximum on day 10, and decreased again. This increase was the most pronounced in the control sample (maximum pH = 7.32). The sharp increase of pH could be an effect of protein oxidation, and the production of ammonia (by utilization of amino acids by spoilage microorganisms) as storage progresses [9]. The initial level of pH in the control samples was similar to that reported by [41]. However others [9] observed an increase of pH values of raw chicken meat over storage period. Generally, the addition of extracts decreased pH values when comparing to control sample, and the lowest pH values were observed for the sample containing cardamom (Table 4). Similar observations have also been made by [9] who reported that the addition of clove extract decreased pH values in comparison to control samples. The effect was ascribed to the antimicrobial activity of compounds found in clove extract which could inhibit the growth and proliferation of spoilage microorganisms that metabolize basic nitrogen compounds [9].

Table 4 contains the results of the instrumental color measurements using L*a*b* color space. All color parameters (L*, a*, b*) were significantly affected by the treatments (*p* = 0.00, F test) and storage time (*p* = 0.01, F test). The lightness of all treated samples (except cardamom) was lower than the control one at each day of the analysis. The highest decrease of L* parameter was observed for clove- and black seed-treated chicken meat. This affected the sensory evaluation of color of these meat samples by the panelist (see below Section 3.6). Similar results were obtained by [41] who reported lower L* values of raw chicken meat samples containing acorn when comparing to control sample. However, others [9] reported significant higher lightness of raw chicken samples with clove extracts in comparison to control sample which is contrary to the results obtained in this study.

The significant reduction in redness (a*) of raw chicken meat was observed after the addition of cardamom, bay leaf and black seed (Table 4). Similar effect was reported previously in raw chicken containing acorn [41]. The highest values of a* parameter were noticed for clove-treated sample. During the first days of storage the value of a* parameter increased slightly, peaked on the 3rd day and decreased thereafter with time in all tested samples. The decrease of a* values with time, and the lowest a* values for control sample were reported by [9]. The changes of a* parameter of raw chicken meat could be due to the formation of metmyoglobin as a result of the interaction between products of pigment oxidation and lipid oxidation [44].

The changes in yellowness (b* values) were very small during storage (Table 4). Apart from bay leaf- and caraway-treated samples with unchanged b* values, all other samples showed slight decrease of the parameter with time. Yellowness of raw chicken meat containing allspice and black seed were significantly lower, whereas nutmeg, bay leaf, and cloves significantly higher when comparing to control sample. The effect of natural extract addition on the color parameters depends strongly on the type of the treatment [13].

### 3.5. Micobiological Analysis

The results on the number of microorganisms in all analyzed samples were shown in Table 5. Based on data analysis, significant differences were found in TVC during storage and between treatments. The starting TVC was approximately 5 log cfu/g for all samples with a value of 5.33 log cfu/g for the control one. The amount of microorganisms increases with storage time. Similar results on TVC values in raw chicken patties during storage at 4 °C were reported by others [12,36]. Generally, the TVC for the samples with plant extract addition was lower than the control sample, and bay leaf was the most potent antimicrobial extract (based on ANCOVA).

*Pseudomonas* spp. and *Enterobateriaceae* counts were significantly affected by the time and treatments. The counts increased with increasing time, and except the sample containing cardamom, all other samples showed lower total mean *Pseudomonas* spp. and *Enterobateriaceae* counts than the control sample. The increases of these Gram negative bacteria with time were consistent with the results reported by [9].

LAB is able to grow both in aerobic and anaerobic conditions and is a substantial part of the natural microflora of meat [9]. The factors of both time and treatments had significant effects on LAB count. The initial LAB counts were from 3.50 for caraway to 3.85 log cfu/g for black seed samples. For all samples the LAB counts increased during the whole storage period reaching the highest level for control sample. The lowest total LAB count was reported for caraway sample (4.32 log cfu/g). Among tested extracts, black seed inhibited the growth of LAB to the least extent. Similar values for control sample and raw chicken meat containing clove extract were reported by [9].

### 3.6. Sensory Analysis

**Figure 5:**
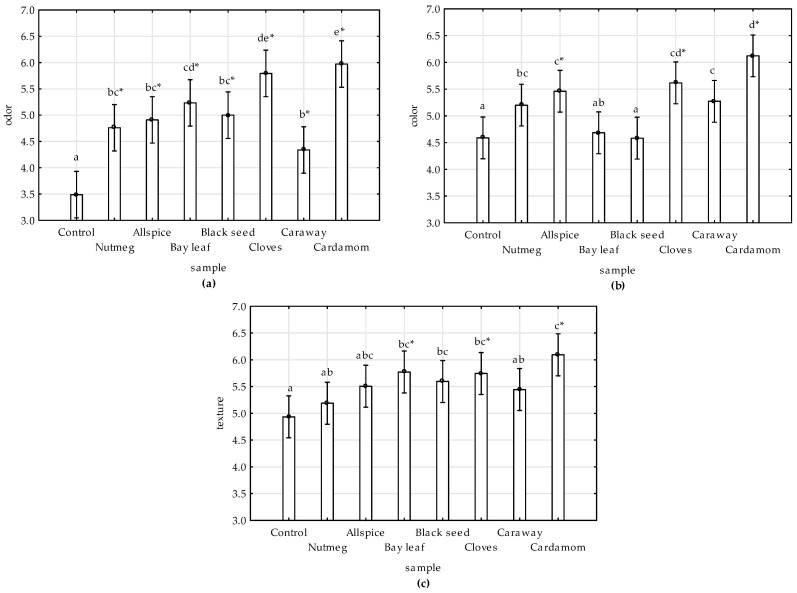
Effect of various spice extract treatments on sensory attributes: (**a**) Odor, (**b**) color, and (**c**) texture of raw ground chicken meat stored at 4 °C during 12 days. Columns present total mean values (from ANCOVA analysis); vertical bars denote 0.95 confidence intervals, and means with the same superscript are not different (*p* > 0.05, Duncan’s test). * indicates significant difference of the sample from the control one (*p* ≤ 0.05, Dunnett’s test).

Results of sensory evaluation (total mean values by ANCOVA) were presented on the Figure 5a–c. Time and treatments had significant effects on sensory scores. The values of all evaluated attributes decreased with time, and the decrease was the most pronounced in the control sample (Figure S1). The highest scores of all attributes were ascribed for the sample with cardamom followed by cloves.

All extracts inhibited the odor alteration of meat during storage when comparing to control (Figure 5a, Figure S1). Similar results were reported by [11], who noticed that sensory scores for control sample differed significantly from pork nuggets with black seed addition, which might result from inhibition of lipid oxidation and off-flavor development during storage by phenolic compounds found in black seed extract. The odor alteration of cooked chicken meat with the addition of wine industry residues extract was also lower comparing to control, as shown by [45]. In accordance to the results of this study, the protective effect of clove extract on odor attribute of raw chicken was also noticed by [9].

In regards to color attribute no significant differences were observed between bay leaf-, black seed-treated, and control samples (Figure 5b). This could result from the dark color of the extracts (Table 4), as was previously observed in chicken meat with grape extract [45].

The addition of spice extract to chicken meat improved the texture of the products (Figure 5c). Others reported lack of statistically significant differences between texture scores of control and black seed-treated pork nuggets [11], and control and plant extract-treated goat meat [46].

### 3.7. Multivariate Analysis

To put more insight into the data, multivariate analyses were performed, namely: PCA, CA, and GDA. Prior to statistical analysis r Pearson’s correlation coefficients were calculated (Table S1) and the data matrix was standardized. Based on the data mining algorithm: Selection and elimination of variables, the following variables were characterized by the highest importance (expressed as Chi-squared) and chosen for further analysis: TBARS, hexanal, CD, and SH contents and L*, a*, b* parameters (Figure S2).

**Figure 6:**
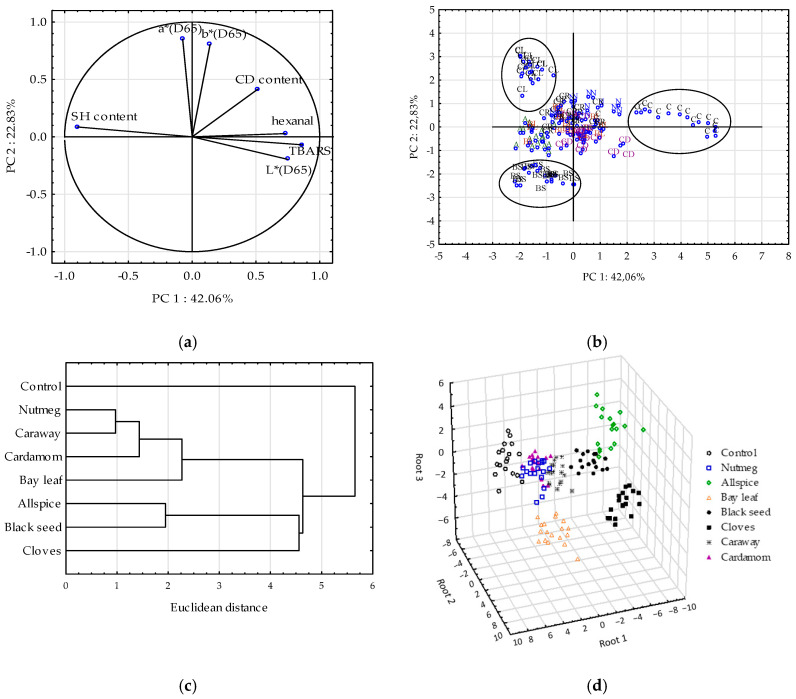
Multivariate analyses: (**a**) Projections of the variables (TBARS—thiobarbituric acid reagent substances, CD—conjugated diene content, SH—thiol group content, hexanal content, L*a*b* color coordinates) onto the factor plane defined by principal components (PC1 and PC2), (**b**) Projections of the scores (samples of raw ground chicken) onto the factor plane (PC1 vs PC2), samples: C—control sample, samples with treatments: A—allspice, BL—bay leaf, BS—black seed, CD—cardamom, CL—cloves, CR—caraway, N—nutmeg, (**c**) Dendrogram of raw ground chicken samples according to CA of similarity, (**d**) GDA classification of raw ground chicken samples.

All parameters of oxidation were significantly correlated (supplementary materials Table S1). The highest r Pearson’s correlation coefficients were determined between SH content and TBARS (−0.70). Significant correlations were also reported between lightness and: Hexanal, TBARS, or SH content. The latter was equal to −0.67. Mir et al. [47] reported in the review that oxidation changes of proteins in chicken meat affected the light scattering and thus L* values. At minimal protein denaturation, low light scattering is observed and the chicken meat has dark color. Thus, it could explain the significant correlation between lightness and protein oxidation observed in the study. The color coordinates: a* and b* were also significantly correlated. The correlations observed were proved by PCA (Figure 6a). PCA gives the possibility to compress the data to a few principal components (PCs) that can be later used to reconstruct data without any preliminary assumptions about their distribution and almost without any loss of information [31,48]. This helps to understand the relationships between variables.

Using graphical criterion, the first four PCs were extracted (three of them with eigenvalue greater than 1) and together they explained 87.3% of the total variance. The PC1 explained 42% of variance and the PC2 the 23% of variance. Based on the loaded values, showing the correlations between components and variables, it could be stated that TBARS, hexanal content, SH content, and lightness (L*) determined sample distribution along PC1 (Figure 6a). Similar results were obtained by [49], who also reported high correlations between lipid, protein and myoglobin oxidation parameters in PCA. Moreover based on the PCA, the authors concluded that myoglobin, lipid, and protein oxidation reactions in rabbit meat were interlocked with each other in a certain manner, and each process seemed to facilitate one another [49]. The same could be stated based on the results showed in this study, since the projection of variable on the factor plane (Figure 6a) indicated complex and inseparable interrelationship among them. When oxidation of lipid occurred the oxidation reactions could be transferred to proteins fraction [40,49] and meat’s pigment [50]. An inverse interaction could be also carried out [51].

Figure 6b showed the projection of the scores on the factor plane. PC1 enabled to separate control sample (on the right of PC1) with the highest lipid oxidation and lightness and the lowest SH content from other samples. Clove-, black seed-, and allspice-treated samples characterized with the lowest protein oxidation (the highest SH groups) among all tested extracts, thus they were on the left of PC1. PC2 was mainly correlated with a* and b* color parameters and it grouped well the samples of raw chicken meat with cloves (with the highest values of a* and b* among samples) and black seed (with the lowest values of a* and b*).

The PCA is less sensitive to differentiate all samples (with allspice, bay leaf, cardamom, caraway and nutmeg). Thus, the CA was applied as another unsupervised pattern recognition technique of multivariate analysis to group samples on the basis of similarities in clusters. The Ward method and Euclidean distance between centroids were applied. The CA dendrogram enabled to separate eight clusters (Figure 6c). All samples with the plant extracts were stacked in one cluster, whereas control sample with high TBARS, hexanal and L* values was stacked in a separate cluster. Moreover, clove-, black seed-, and allspice-treated samples with low lipid/protein oxidation heaped in one cluster different from the rest of treated samples. Allspice and black seed, both with low values of b* coordinates, were stacked in another cluster than cloves showing high b* value. Also bay leaf (with low a* value) was heaped in clusters other than cardamom, caraway, and nutmeg. The lowest Euclidean distance was measured between nutmeg- and caraway-treated samples. These samples were the most similar according to the tested parameters, which disrupted to discriminate them well. The small distance was also noticed between caraway and cardamom samples.

GDA is supervised pattern recognition technique enabling obtainment of discriminant functions, which maximize the ratio of between-class variance and minimize the ratio of within-class variance [31]. The first 5 discriminant functions were statistically significant and explained 99.9% of total variance. The first discriminant function accounted for 49.7% of total variance, and the second one for 33.6%. Based on standardized canonical discriminant function coefficients the importance of the independent variables in the canonical function was discussed (Table 6). The higher the absolute value of standardized coefficient the higher the discrimination power of the related variable is.

SH content contributed the most to the first canonical function, CD content to the second one, whereas a* parameter to the third one. The discrimination of meat samples with spice extracts was noticeable (Figure 6d). Based on the results it could be concluded that meat samples showing very high oxidative stability of proteins, namely: Clove-, allspice-, and black seed-treated samples, were excellent discriminated with 100% of the classification’s propriety. The propriety of classification was also 100% for the samples treated with bay leaf and caraway. Only 2 control samples, 3 samples with nutmeg, and 2 samples with cardamom extracts were incorrectly classified. Summarizing, according to the classification matrix 95.1% of the samples were classified correctly which proved very strong classification ability of the constructed discrimination models. Discriminant functions were successfully used previously to classified samples of raw ground pork with garlic and marjoram according to the fatty acid composition [10].

## 4. Conclusions

The results of this study indicated clearly that the addition of spice extracts decreased lipid and protein oxidation and increased microbiological stability of raw ground chicken stored at 4 °C. In order to eliminate incomplete or false conclusions on the antioxidant effectiveness of spice extracts various methods for oxidation measurements were combined. In this study, cloves, allspice, and black seed were the most potent antioxidant active spices among tested ones. They also maintained microbiological quality of chicken meat, as it was shown based on TVC count. While the beneficial effects of clove and allspice extracts could be attributed to the high antioxidant activity, the protective influence of black seed on raw chicken meat quality did not correlate with the radical scavenging activity. In spite of having enormous medicinal importance hardly any study had been conducted to investigate the efficiency of black seed in muscle food system. The present study showed for the first time that the extract of black seed could be used to extend the shelf-life of raw ground chicken meat very effectively. However, meat sample with black seed was assessed very low in sensory analysis (color). Moreover, chemometric techniques could put more insight into the data structure. Based on PCA analysis it could be stated that each oxidation process (lipid, protein) could contribute to the initiation of another one and that L* color coordinate of meat was highly correlated to the oxidation of protein in meat. Finally, according to the multivariate analyses the noticeable discrimination of samples was performed based on the oxidation parameters and color coordinates, showing a great impact of extracts on the final quality of raw chicken meat.

## Figures and Tables

**Table 1:** ABTS^•+^ radical scavenging activity of spice extracts.

Extracts	TEAC (ABTS) µM/g
Nutmeg	44.80 ± 1.93 ^d^
Allspice	815.37 ± 20.85 ^b^
Bay Leaf	280.03 ± 21.51 ^c^
Black seed	33.06 ± 2.79 ^de^
Cloves	2495.85 ± 31.41 ^a^
Caraway	35.37 ± 0.39 ^de^
Cardamom	12.93 ± 0.46 ^e^

All values are mean ± SD of the three replicates. ^(a–e)^ means with the same superscript are not different (*p* > 0.05, Duncan’s test).

**Table 2:** UPLC analysis of phenolic compounds in spice extracts (mg/kg DW).

Phenolic Compounds	Nutmeg	Allspice	Bay Leaf	Black Seed	Cloves	Caraway	Cardamom
protocatechuic acid	0.23 ^a^	0.36 ^a^	<LOD	1.26 ^a^	79.57 ^c^	0.22 ^a^	12.57 ^b^
2,5-dihydroxybenzoic acid	<LOD	<LOD	16.52 ^d^	1.23 ^bc^	1.33 ^c^	<LOD	0.23 ^ab^
4-hyrdoxybenzoic acid	0.19 ^a^	<LOD	36.50 ^c^	<LOD	40.12 ^d^	10.69 ^b^	2.36 ^a^
gallic acid	10.24 ^c^	<LOD	<LOD	<LOD	0.36 ^a^	0.16 ^a^	1.39 ^b^
syryngic acid	10.84 ^c^	0.75 ^ab^	0.66 ^ab^	12.66 ^d^	1.22 ^b^	<LOD	<LOD
t-cinnamic acid	3.95 ^b^	0.00	19.62 ^e^	<LOD	29.50 ^d^	6.58 ^c^	1.49 ^a^
caffeic acid	2.30 ^a^	<LOD	<LOD	<LOD	52.90 ^b^	59.30 ^c^	<LOD
p-coumaric acid	<LOD	<LOD	<LOD	29.54 ^b^	5.30 ^a^	<LOD	<LOD
ferulic acid	<LOD	<LOD	0.23 ^a^	<LOD	2.10 ^b^	<LOD	<LOD
sinapic acid	0.22 ^ab^	0.06 ^a^	1.95 ^d^	3.45 ^e^	<LOD	0.59 ^b^	1.46 ^c^
chlorogenic acid	0.06 ^a^	0.49 ^a^	0.56 ^a^	0.37 ^a^	10.20 ^b^	0.11 ^a^	0.46 ^a^
total phenolic acids	27.97 ^b^	1.16 ^a^	75.47 ^d^	48.14 ^c^	212.4 ^e^	77.52 ^d^	19.50 ^b^
eriodictyol	0.26 ^a^	<LOD	0.45 ^b^	<LOD	<LOD	1.13 ^c^	0.22 ^a^
apigenin	12.83 ^c^	0.36 ^a^	12.65 ^c^	<LOD	0.07 ^a^	0.06 ^a^	6.33 ^b^
luteolin	0.23 ^a^	0.36 ^a^	10.54 ^b^	19.50 ^c^	0.06 ^a^	12.36 ^b^	27.50 ^d^
catechin	57.20 ^b^	0.23 ^a^	1.26 ^a^	<LOD	0.39 ^a^	<LOD	1.68 ^a^
rutin	<LOD	7.25 ^c^	1.95 ^b^	0.13 ^a^	<LOD	<LOD	<LOD
kaempferol	0.44 ^a^	10.65 ^c^	0.11 ^a^	4.52 ^b^	0.05 ^a^	0.26 ^a^	<LOD

All values are mean of the three replicates. ^(a–e)^ means with the same superscript within the same row are not different (*p* > 0.05, Duncans’s test). LOD—limit of detection.

**Table 3:** Effect of various spice extract treatments on hexanal content (mg/100 g meat) in raw ground chicken legs during 12 days of refrigerated storage (4 °C).

Storage Days	Control	Nutmeg	Allspice	Bay Leaf	Black Seed	Cloves	Caraway	Cardamom
0	10.13 ± 0.83 ^aA^	5.51 ± 0.23 ^cC^	3.84 ± 0.36 ^dB^	3.17 ± 0.77 ^dA^	1.69 ± 0.20 ^eA^	0.02 ± 0.01 ^fA^	3.83 ± 0.26 ^dAB^	6.69 ± 0.47 ^bB^
3	17.20 ± 1.37 ^aB^	7.45 ± 0.28 ^dD^	5.99 ± 0.26 ^dC^	9.66 ± 0.95 ^cB^	2.29 ± 0.35 ^eAB^	0.16 ± 0.01 ^fB^	7.52 ± 1.64 ^dC^	11.71 ± 1.28 ^bC^
5	19.28 ± 1.20 ^aB^	17.85 ± 1.52 ^aE^	0.32 ± 0.05 ^eA^	13.06 ± 2.46 ^bB^	3.14 ± 0.25 ^dBC^	nd ± nd	5.63 ± 2.05 ^cBC^	11.07 ± 1.49 ^bC^
7	28.24 ± 2.62 ^aC^	20.93 ± 0.53 ^bF^	nd ± nd	21.29 ± 4.36 ^bC^	3.74 ± 0.29 ^dC^	nd ± nd	3.36 ± 0.89 ^deA^	8.68 ± 1.96 ^cB^
10	39.17 ± 4.90 ^aD^	2.27 ± 0.64 ^bcB^	nd ± nd	nd ± nd	4.60 ± 0.35 ^bD^	nd ± nd	nd ± nd	0.85 ± 0.05 ^cA^
12	9.63 ± 0.81 ^aA^	0.62 ± 0.20 ^cA^	nd ± nd	nd ± nd	6.01 ± 0.98 ^bE^	nd ± nd	nd ± nd	0.33 ± 0.21 ^cA^

All values are mean ± SD of the three replicates; nd—not detected;^(a–f)^ means with the same superscript within the same row are not different (*p* > 0.05, Duncan’s test)—effect of treatments; (A–F) means with the same superscript within the same column are not different (*p* > 0.05, Duncan’s test)—effect of time.

**Table 4:** Effect of various spice extract treatments on pH values and color coordinates (L*a*b*) of raw ground chicken legs during 12 days of refrigerated storage (4 °C).

Storage Days	Control	Nutmeg	Allspice	Bay Leaf	Black Seed	Cloves	Caraway	Cardamom
**pH**								
0	6.33 ± 0.01 ^cB^	6.35 ± 0.00 ^cD^	6.25 ± 0.01 ^bB^	6.35 ± 0.00 ^cBC^	6.24 ± 0.02 ^bB^	6.26 ± 0.00 ^bA^	6.33 ± 0.02 ^cB^	6.20 ± 0.02 ^aC^
3	6.24 ± 0.01 ^cAB^	6.24 ± 0.02 ^cCD^	6.21 ± 0.01 ^bAB^	6.25 ± 0.00 ^cAB^	6.14 ± 0.01 ^aAB^	6.21 ± 0.01 ^bA^	6.24 ± 0.02 ^cAB^	6.12 ± 0.02 ^aB^
5	6.10 ± 0.03 ^aA^	6.19 ± 0.06 ^bBC^	6.16 ± 0.02 ^bAB^	6.18 ± 0.02 ^bAB^	6.14 ± 0.01 ^abAB^	6.14 ± 0.02 ^abA^	6.19 ± 0.02 ^bAB^	6.10 ± 0.01 ^aB^
7	6.17 ± 0.07 ^cAB^	6.02 ± 0.01 ^aA^	6.07 ± 0.01 ^abA^	6.12 ± 0.03 ^bcA^	6.09 ± 0.03 ^bA^	6.09 ± 0.02 ^bA^	6.13 ± 0.01 ^bcA^	6.03 ± 0.00 ^aA^
10	7.32 ± 0.22 ^cC^	6.70 ± 0.17 ^bE^	6.75 ± 0.15 ^bC^	6.51 ± 0.18 ^abC^	6.67 ± 0.17 ^bC^	6.70 ± 0.21 ^bB^	6.82 ± 0.12 ^bD^	6.35 ± 0.03 ^aD^
12	6.32 ± 0.11 ^cdB^	6.08 ± 0.01 ^aAB^	6.31 ± 0.16 ^bcdB^	6.26 ± 0.14 ^abcAB^	6.10 ± 0.01 ^aA^	6.20 ± 0.09 ^abcA^	6.49 ± 0.16 ^dC^	6.11 ± 0.03 ^abB^
L*								
0	62.95 ± 0.75 ^eA^	60.26 ± 0.49 ^cdA^	58.95 ± 1.20 ^bcA^	57.78 ± 1.09 ^abA^	57.02 ± 0.19 ^aA^	56.40 ± 0.78 ^aC^	59.16 ± 1.03 ^bcA^	61.18 ± 0.89 ^dA^
3	63.55 ± 0.61 ^gA^	61.10 ± 0.24 ^efAB^	59.19 ± 1.15 ^cdA^	58.43 ± 0.99 ^bcA^	57.66 ± 0.13 ^abAB^	56.54 ± 0.56 ^aC^	60.33 ± 0.91 ^deA^	62.01 ± 0.96 ^fA^
5	63.40 ± 0.33 ^fA^	61.14 ± 0.43 ^dB^	58.99 ± 1.16 ^cA^	58.65 ± 0.72 ^cA^	57.48 ± 0.29 ^bBC^	56.30 ± 0.49 ^aC^	60.24 ± 0.68 ^deA^	62.09 ± 0.70 ^eA^
7	62.96 ± 0.52 ^eA^	60.95 ± 0.45 ^dAB^	58.37 ± 0.79 ^bcA^	58.98 ± 0.59 ^cA^	57.53 ± 0.38 ^bAB^	55.46 ± 0.53 ^aBC^	60.20 ± 0.69 ^dA^	62.11 ± 0.50 ^eA^
10	62.69 ± 0.74 ^fA^	60.99 ± 0.54 ^deAB^	57.45 ± 0.88 ^bA^	58.91 ± 0.63 ^cA^	58.05 ± 0.22 ^bcC^	54.59 ± 0.66 ^aAB^	60.29 ± 0.76 ^dA^	61.82 ± 0.47 ^efA^
12	62.50 ± 0.92 ^eA^	61.11 ± 0.39 ^dAB^	57.33 ± 0.84 ^bA^	59.27 ± 0.49 ^cA^	58.61 ± 0.40 ^cD^	54.34 ± 0.36 ^aA^	60.64 ± 0.66 ^dA^	61.70 ± 0.43 ^deA^
a*								
0	4.42 ± 0.20 ^deC^	4.98 ± 0.26 ^eB^	3.75 ± 0.39 ^cdA^	2.11 ± 0.27 ^bAB^	1.29 ± 0.20 ^aA^	6.07 ± 0.42 ^fA^	4.38 ± 0.57 ^deAB^	3.35 ± 0.57 ^cAB^
3	6.21 ± 0.21 ^efE^	6.68 ± 0.20 ^fgD^	5.41 ± 0.32 ^cdD^	3.54 ± 0.71 ^bC^	2.41 ± 0.30 ^aC^	7.35 ± 0.36 ^gD^	5.90 ± 0.66 ^cdeC^	5.26 ± 0.72 ^cC^
5	5.60 ± 0.08 ^cdD^	6.46 ± 0.16 ^eD^	5.21 ± 0.39 ^cdD^	3.26 ± 0.61 ^bC^	2.40 ± 0.22 ^aC^	7.28 ± 0.29 ^fCD^	5.77 ± 0.55 ^deC^	4.92 ± 0.66 ^cC^
7	4.76 ± 0.13 ^cdC^	5.90 ± 0.15 ^eC^	4.92 ± 0.17 ^cdCD^	2.79 ± 0.53 ^bBC^	1.97 ± 0.17 ^aB^	7.15 ± 0.36 ^fCD^	5.28 ± 0.69 ^deBC^	4.29 ± 0.49 ^cBC^
10	3.40 ± 0.27 ^bB^	5.05 ± 0.08 ^cB^	4.59 ± 0.26 ^cBC^	1.98 ± 0.40 ^aAB^	1.36 ± 0.24 ^aA^	6.70 ± 0.34 ^dBC^	4.39 ± 0.82 ^cAB^	3.18 ± 0.55 ^bA^
12	2.85 ± 0.36 ^bA^	4.36 ± 0.15 ^cA^	4.30 ± 0.27 ^cB^	1.51 ± 0.42 ^aA^	0.96 ± 0.19 ^aA^	6.45 ± 0.13 ^dAB^	3.74 ± 0.66 ^cA^	2.77 ± 0.47 ^bA^
b*								
0	13.11 ± 0.46 ^bcA^	13.87 ± 0.30 ^cdC^	10.84 ± 0.80 ^aA^	14.14 ± 0.46 ^dA^	10.15 ± 0.18 ^aA^	14.49 ± 0.97 ^dAB^	12.85 ± 0.37 ^bA^	13.03 ± 0.14 ^bcD^
3	13.03 ± 0.38 ^bcA^	13.62 ± 0.18 ^cdBC^	10.98 ± 0.70 ^aA^	13.98 ± 0.34 ^dA^	10.55 ± 0.10 ^aCD^	15.01 ± 0.65 ^eB^	12.89 ± 0.33 ^bcA^	12.76 ± 0.12 ^bD^
5	13.04 ± 0.44 ^bA^	13.50 ± 0.24 ^bcBC^	10.95 ± 1.04 ^aA^	13.95 ± 0.31 ^cdA^	10.59 ± 0.23 ^aD^	14.61 ± 0.45 ^dAB^	13.18 ± 0.34 ^bcA^	12.84 ± 0.18 ^bD^
7	12.75 ± 0.46 ^bA^	13.14 ± 0.32 ^bcAB^	10.76 ± 0.78 ^aA^	13.73 ± 0.30 ^cdA^	10.26 ± 0.13 ^aAB^	14.07 ± 0.53 ^dAB^	13.11 ± 0.36 ^bcA^	12.53 ± 0.10 ^bBC^
10	12.68 ± 0.35 ^bcA^	13.15 ± 0.37 ^cdAB^	10.54 ± 0.55 ^aA^	13.93 ± 0.15 ^eA^	10.19 ± 0.29 ^aAB^	13.84 ± 0.53 ^deA^	13.26 ± 0.42 ^cdeA^	12.46 ± 0.16 ^bAB^
12	12.54 ± 0.29 ^bcA^	12.74 ± 0.25 ^cdA^	10.11 ± 0.31 ^aA^	13.77 ± 0.20 ^fA^	9.93 ± 0.24 ^aA^	13.46 ± 0.16 ^efA^	13.08 ± 0.24 ^deA^	12.22 ± 0.23 ^bA^

All values are mean ± SD of the three replicates. (a–g) means with the same superscript within the same row are not different (*p* > 0.05, Duncan’s test)—effect of treatments; (A–E) means with the same superscript within the same column are not different (*p* > 0.05, Duncan’s test)—effect of time.

**Table 5:** Effect of various spice extract treatments on microbiological quality expressed as TVC—total viable count, *Psedomonas*, *Enterobactericeae,* and LAB—lactic acid bacteria of raw ground chicken legs during 12 days of refrigerated storage (4 °C).

Storage Days	Control	Nutmeg	Allspice	Bay Leaf	Black Seed	Cloves	Caraway	Cardamom
**Total Viable Count (TVC) log cfu/g Meat**								
0	5.33 ± 0.35 ^bcA^	5.23 ± 0.22 ^bcA^	4.89 ± 0.28 ^aA^	5.44 ± 0.34 ^cA^	5.11 ± 0.27 ^abA^	5.09 ± 0.25 ^abA^	5.27 ± 0.18 ^bcA^	5.26 ± 0.13 ^bcA^
3	6.94 ± 0.10 ^dB^	6.38 ± 0.20 ^aB^	6.83 ± 0.11 ^cdB^	6.66 ± 0.19 ^bcB^	7.25 ± 0.43 ^eB^	6.60 ± 0.18 ^bB^	6.99 ± 0.19 ^dB^	6.87 ± 0.04 ^cdB^
5	8.57 ± 0.14 ^cdC^	8.28 ± 0.40 ^abcC^	8.29 ± 0.26 ^abcC^	8.51 ± 0.18 ^bcdC^	8.25 ± 0.22 ^abCc^	8.09 ± 0.10 ^aC^	8.21 ± 0.57 ^abC^	8.62 ± 0.20 ^dC^
7	9.59 ± 0.19 ^eD^	9.38 ± 0.36^cdeD^	9.53 ± 0.26 ^deD^	8.65 ± 0.23 ^aC^	8.96 ± 0.07 ^bD^	9.24 ± 0.20 ^bcdD^	9.16 ± 0.37 ^bcD^	9.25 ± 0.49 ^bcdD^
10	9.64 ± 0.15 ^bD^	9.49 ± 0.22 ^abD^	9.67 ± 0.19 ^bDE^	9.11 ± 0.66 ^aD^	9.15 ± 0.33 ^aD^	9.45 ± 0.41 ^abD^	9.51 ± 0.16 ^abE^	9.65 ± 0.56 ^bE^
12	9.78 ± 0.10 ^aD^	9.56 ± 0.30 ^aD^	9.93 ± 0.55 ^aE^	9.54 ± 0.42 ^aE^	9.56 ± 0.31 ^aE^	9.70 ± 1.05 ^aD^	9.86 ± 0.11 ^aF^	9.99 ± 0.67 ^aE^
***Pseudomonas* log cfu/g Meat**								
0	5.38 ± 0.21 ^abA^	5.24 ± 0.16 ^abA^	5.11 ± 0.23 ^aA^	5.31 ± 0.18 ^abA^	5.34 ± 0.16 ^abA^	5.39 ± 0.18 ^bA^	5.46 ± 0.35 ^bA^	5.48 ± 0.38 ^aA^
3	7.44 ± 0.07 ^cB^	7.09 ± 0.38 ^bB^	7.47 ± 0.12 ^cB^	7.33 ± 0.08 ^cB^	7.31 ± 0.04 ^cB^	6.83 ± 0.14 ^aB^	7.33 ± 0.07 ^cB^	7.40 ± 0.11 ^cB^
5	8.96 ± 0.18 ^cC^	8.31 ± 0.05 ^aC^	8.34 ± 0.17 ^aC^	8.35 ± 0.64 ^aC^	8.67 ± 0.21 ^bcC^	8.29 ± 0.24 ^aC^	8.44 ± 0.19 ^abC^	8.74 ± 0.15 ^cC^
7	10.25 ± 0.15 ^cD^	9.21 ± 0.09 ^abD^	9.14 ± 0.24 ^abD^	9.50 ± 0.57 ^bD^	9.21 ± 0.51 ^abD^	9.25 ± 0.65 ^abD^	9.07 ± 0.09 ^aD^	9.89 ± 0.07 ^cD^
10	10.32 ± 0.18 ^cD^	9.50 ± 0.32 ^aE^	9.31 ± 0.32 ^aD^	9.55 ± 0.55 ^aD^	9.57 ± 0.33 ^aE^	9.30 ± 0.17 ^aD^	9.44 ± 0.21 ^aE^	9.95 ± 0.07 ^bD^
12	10.48 ± 0.09 ^dE^	10.07 ± 0.20 ^cF^	9.77 ± 0.33 ^abE^	9.62 ± 0.22 ^aD^	9.72 ± 0.46 ^abE^	9.75 ± 0.27 ^abE^	10.00 ± 0.09 ^bcF^	10.20 ± 0.15 ^cE^
***Enterobacteriaceae* log cfu/g Meat**								
0	3.96 ± 0.09 ^eA^	3.49 ± 0.10 ^bA^	3.84 ± 0.06 ^cdA^	3.35 ± 0.12 ^aA^	3.77 ± 0.16 ^cA^	3.58 ± 0.08 ^bA^	3.38 ± 0.12 ^aA^	3.89 ± 0.07 ^deA^
3	5.01 ± 0.11 ^bB^	4.90 ± 0.14 ^bB^	4.95 ± 0.11 ^bB^	5.12 ± 0.09 ^bB^	5.00 ± 0.07 ^bB^	4.97 ± 0.10 ^bB^	4.59 ± 0.53 ^aB^	4.98 ± 0.13 ^bB^
5	6.52 ± 0.10 ^eC^	6.00 ± 0.08 ^bC^	6.21 ± 0.12 ^cC^	6.44 ± 0.07 ^eC^	6.07 ± 0.03 ^bC^	5.85 ± 0.27 ^aC^	6.42 ± 0.11 ^deC^	6.30 ± 0.04 ^cdC^
7	7.62 ± 0.17 ^dD^	7.09 ± 0.31 ^aD^	7.24 ± 0.16 ^abD^	7.33 ± 0.06 ^abcD^	7.14 ± 0.49 ^aD^	7.40 ± 0.09 ^bcdD^	7.51 ± 0.12 ^cdD^	7.42 ± 0.11 ^bcdD^
10	7.83 ± 0.09 ^dE^	7.34 ± 0.15 ^aE^	7.45 ± 0.11 ^abcE^	7.54 ± 0.19 ^bcE^	7.38 ± 0.20 ^abE^	7.50 ± 0.08 ^abcD^	7.59 ± 0.15 ^cD^	7.51 ± 0.13 ^bcD^
12	8.05 ± 0.37 ^bF^	7.51 ± 0.22 ^aE^	7.60 ± 0.26 ^aE^	7.66 ± 0.20 ^aE^	7.46 ± 0.15 ^aE^	7.56 ± 0.44 ^aD^	7.60 ± 0.17 ^aD^	7.66 ± 0.17 ^aE^
**Lactic Acid Bacteria (LAB) log cfu/g Meat**								
0	3.73 ± 0.08 ^deA^	3.76 ± 0.12 ^deA^	3.69 ± 0.17 ^cdA^	3.55 ± 0.13 ^abA^	3.85 ± 0.10 ^eA^	3.59 ± 0.12 ^abcA^	3.50 ± 0.15 ^aA^	3.64 ± 0.11 ^bcdA^
3	4.43 ± 0.12 ^dB^	3.81 ± 0.12 ^bA^	4.43 ± 0.19 ^dB^	3.88 ± 0.10 ^bcB^	4.36 ± 0.10 ^dB^	3.53 ± 0.18 ^aA^	3.50 ± 0.14 ^aA^	3.95 ± 0.10 ^cB^
5	4.64 ± 0.18 ^dC^	4.46 ± 0.15 ^cB^	4.55 ± 0.13 ^cdB^	4.46 ± 0.11 ^cC^	4.51 ± 0.09 ^cdB^	3.93 ± 0.15 ^aB^	4.14 ± 0.18 ^bB^	4.50 ± 0.07 ^cdC^
7	5.52 ± 0.15 ^dD^	5.20 ± 0.13 ^cC^	5.05 ± 0.13 ^bcC^	5.12 ± 0.10 ^cD^	5.20 ± 0.18 ^cC^	5.55 ± 0.23 ^dC^	4.75 ± 0.14 ^aC^	4.90 ± 0.20 ^abD^
10	5.61 ± 0.32 ^deD^	5.26 ± 0.18 ^bcC^	5.19 ± 0.12 ^abC^	5.47 ± 0.41 ^cdeE^	5.37 ± 0.13 ^bcdC^	5.67 ± 0.20 ^eCD^	4.95 ± 0.30 ^aD^	5.38 ± 0.09 ^bcdE^
12	5.93 ± 0.09 ^fE^	5.50 ± 0.22 ^bcD^	5.41 ± 0.16 ^bD^	5.70 ± 0.24 ^cdeF^	5.72 ± 0.30 ^deD^	5.81 ± 0.05 ^efD^	5.07 ± 0.15 ^aD^	5.58 ± 0.26 ^bcdF^

All values are mean ± SD of the three replicates. (a–f) means with the same superscript within the same row are not different (*p* > 0.05, Duncan’s test)—effect of treatments; (A–E) means with the same superscript within the same column are not different (*p* > 0.05, Duncan’s test)—effect of time.

**Table 6:** Standardized canonical discriminant function coefficients.

	Root 1	Root 2	Root 3	Root 4	Root 5
TBARS	0.192	−0.193	0.110	−0.219	0.423
hexanal content	0.334	−0.209	−0.320	0.716	−0.151
CD content	−0.569	1.150	0.633	0.637	0.987
SH content	−1.050	0.516	0.320	−0.148	1.414
L*(D65)	0.577	−0.198	0.499	−0.235	0.532
a*(D65)	−0.076	0.430	1.029	−0.174	−0.442
b*(D65)	0.736	0.689	−0.574	−0.119	0.075
discrimination %	49.68%	33.52%	12.71%	2.60%	1.35%
cumulative %	49.68%	83.20%	95.91%	98.51%	99.86%

## Supplementary Materials

The following are available online at https://www.mdpi.com/2076-3921/9/9/903/s1, Figure S1: Effects of time and treatments on sensory attributes; Figure S2: Importance (expressed as Chi-squared value) of the predictive variables; Table S1: r Pearson’s correlation coefficients between parameters of lipid and protein oxidation.
