# Supplementary material for: A Chemometric Approach to Oxidative Stability and Physicochemical Quality of Raw Ground Chicken Meat Affected by Black Seed and Other Spice Extracts
Source: Antioxidants (Basel). 2020 Sep 22;9(9):903. doi: 10.3390/antiox9090903 (PMC7555872; doi:10.3390/antiox9090903)
Supplement: Supplementary file 1 [file antioxidants-09-00903-s001.pdf]

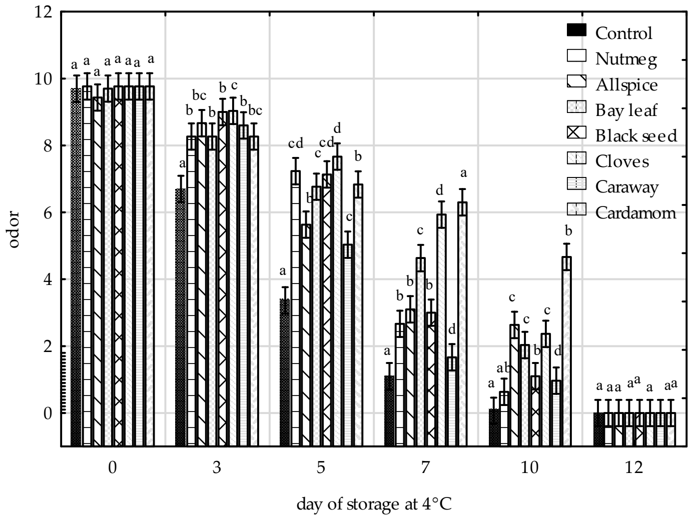


**(a)**

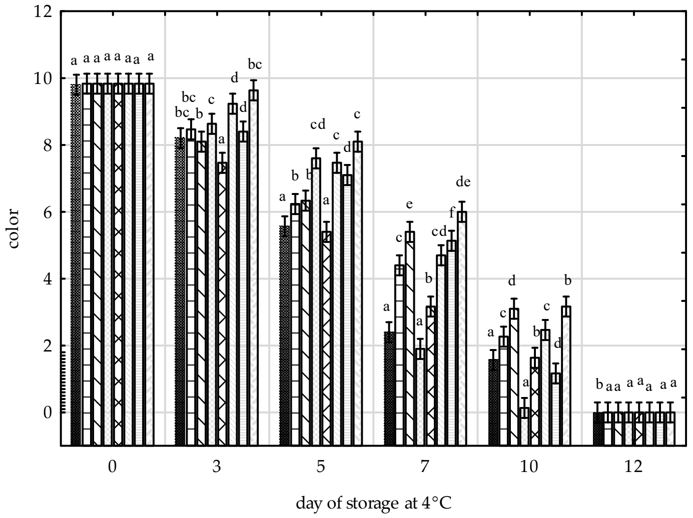


**(b)**

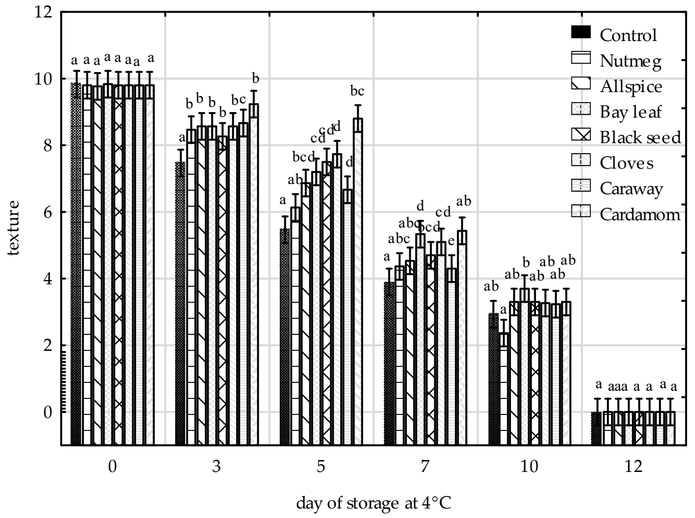


**(c)**

**Figure S1.** Effects of time and treatments on sensory attributes: (a) odor, (b) color, (c) texture of raw ground chicken meat stored at 4°C during 12 days. Columns present total mean values; vertical bars denote 0.95 confidence intervals, and means with the same superscript are not different ($p > 0.05$, Duncan's test).

**Table S1.** r Pearson's correlation coefficients between parameters of lipid and protein oxidation (TBARS – thiobarbituric acid reagent substances, CD – conjugated diene content, SH – thiol group content, hexanal content), and color coordinates (L*a*b*). * statistically significant coefficient ($p \leq 0.05$, Student's t test).

| | TBARS | hexanal | CD content | SH content | L*(D65) | a*(D65) | b*(D65) |
|---|---|---|---|---|---|---|---|
| **TBARS** | 1.00 | | | | | | |
| **hexanal** | 0.54* | 1.00 | | | | | |
| **CD content** | 0.46* | 0.17* | 1.00 | | | | |
| **SH content** | -0.70* | -0.49* | -0.53* | 1.00 | | | |
| **L*(D65)** | 0.52* | 0.55* | -0.02 | -0.67* | 1.00 | | |
| **a*(D65)** | -0.15 | 0.04 | 0.19* | 0.17* | -0.07 | 1.00 | |
| **b*(D65)** | 0.06 | 0.12 | 0.19* | -0.05 | 0.03 | 0.48* | 1.00 |

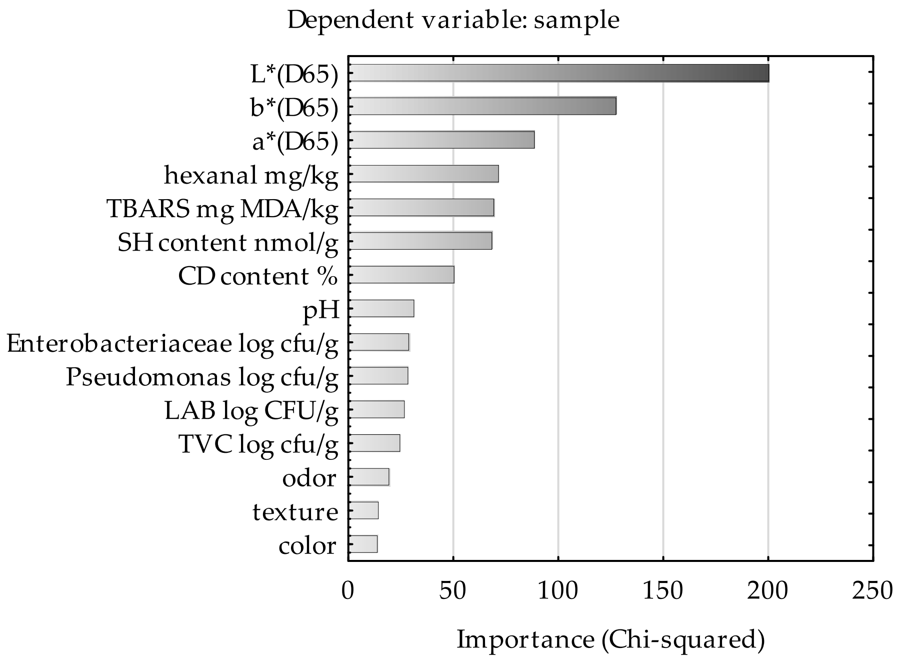


**Figure S2.** Importance (expressed as Chi-squared value) of the predictive variables (TBARS – thiobarbituric acid reagent substances, CD – conjugated diene content, SH – thiol group content, hexanal content, pH, color coordinates: L*a*b*, TVC – total viable count, *Psedomonas*, *Enterobactericeae* and LAB – lactic acid bacteria, odor, color, texture) on the dependent variable: sample.
